# OA29 Blotting-based assays under-detected TIF1-gamma and NXP2 autoantibodies compared to immunoprecipitation in Juvenile Dermatomyositis cohorts

**DOI:** 10.1093/rap/rkac066.029

**Published:** 2022-09-28

**Authors:** Huong D Nguyen, Charalampia Papadopoulou, Dario Cancemi, Lucy R Wedderburn, Sarah L Tansley

**Affiliations:** UCL Great Ormond Street Institute of Child Health, London, United Kingdom; NIHR Biomedical Research Centre at Great Ormond Street Hospital for Children, London, United Kingdom; UCL Great Ormond Street Institute of Child Health, London, United Kingdom; UCL Great Ormond Street Institute of Child Health, London, United Kingdom; NIHR Biomedical Research Centre at Great Ormond Street Hospital for Children, London, United Kingdom; Centre for Adolescent Rheumatology Versus Arthritis at UCL, UCLH and GOSH, London, United Kingdom; Royal United Hospitals Bath NHS Foundation Trust, Bath, United Kingdom; The University of Bath, Bath, United Kingdom

## Abstract

**Introduction/Background:**

The rare heterogenous autoimmune disease Juvenile Dermatomyositis (JDM) relies on prognostic tools including myositis specific antibodies (MSA) and myositis associated antibodies (MAA) to guide early treatment and reduce the risks of poor outcome. However, there is an emerging concern regarding the inter-assay validity due to the current lack of a standardised protocol for MSA/MAA screening across healthcare centres. Among available MSA/MAA screening assays, most laboratories prefer using immunoblot over immunoprecipitation, due to its practicality and lower cost. Notably, blotting-based assays were reported to have reduced specificity at detecting certain MSA/MAA subgroups.

**Description/Method:**

A total of 472 JDM patients recruited via UK Juvenile Dermatomyositis Cohort and Biomarker Study (JDCBS) were included in this study. For immunoprecipitation cohort, the sera of 383 JDM-diagnosed patients were determined at the University of Bath using radio-labelled protein immunoprecipitation, and 140kDa bands were confirmed with ELISA for NXP2 or MDA5. For immunoblot cohort (n = 89), MSA/MAA results were obtained from the patients’ records at the centre of care. The immunoprecipitation group and immunoblot group were investigated for the frequency of each autoantibody subgroup within each cohort. The objective was to determine the sensitivity of each assay in detecting myositis relevant antibodies (MSA/MAA) in the context of JDM.

**Discussion/Results:**

Overall, the majority of patients were female, and self-identified as white. Specifically, in immunoprecipitation cohort, 70.9% of the patients were female, while 77.1% of the patients self-identified as white. In the immunoblot cohort, 72.7% of the patient were female, while 65.9% self-identified as white.

Regarding MSA/MAA status in each cohort, 225 out of 383 (58%) immunoprecipitation samples were positive for at least 1 myositis relevant autoantibody (MSA/MAA). In the immunoblot cohort the proportion was 53% (47 out of 89 samples). Despite the similar gender and ethnicity distribution, the frequencies of autoantibody subgroups varied between two cohorts. In descending order, the 3 most common MSAs detected by immunoprecipitation group were anti-TIF1γ (18.1%), anti-NXP2 (15.8%) and anti-MDA5 (5.9%). On the contrary, anti-MDA5 (15.5%) was the most prevalent autoantibody detected by immunoblot, followed by anti-TIF1γ (11.3%) and anti-NXP2 (9.3%), respectively.

The prevalence of more than one myositis relevant autoantibodies detected in a patient was rare in both cohorts. Specifically, when excluding anti-Ro52, a MAA that is not detected with immunoprecipitation, 2.2% of the total patients in immunoblot cohort had more than 1 MSA/MAA. This rate is lower in immunoprecipitation cohort, as only 0.5% of the patients in this cohort were determined to be positive for more than one autoantibody subgroup. When accounting for anti-Ro52 in immunoblot cohort, 7 out of 8 anti-Ro52 positive patients were also positive for anti-MDA5, making up 7.9% of immunoblot cohort.

**Key learning points/Conclusion:**

The variance between autoantibody frequencies detected by each method suggested that immunoprecipitation and immunoblot assays do not perform equally for all MSA and MAA. The lower detection of anti-TIF1γ in immunoblot is consistent with previous reports. Additionally, a potential reduction of sensitivity in anti-NXP2 detection using immunoblot is observed for the first time. Higher rate of anti-MDA5 in immunoblot cohort might reflect the earlier pre-treatment samples compared to the samples tested in immunoprecipitation cohort, as the titre of anti-MDA5 was reported to decrease with treatment. As MSA/MAA status are interpreted by clinicians to classify JDM patients into homogenous subgroups for more focused treatment plans, the reliability of autoantibody screening process is an issue of interest. Further investigations are necessary to characterise the sensitivity and specificity of each method.

